# Passively Q-Switched Yb:CALGO Laser Based on Mo:BiVO_4_ Absorber

**DOI:** 10.3390/nano11092364

**Published:** 2021-09-11

**Authors:** Lina Zhao, Ye Yuan, Luyang Tong, Wenyu Zhang, Zhongshuai Zhang, Tingyin Ning, Yangjian Cai, Yuanmei Gao

**Affiliations:** 1 College of Physics and Electronics, Center of Light Manipulations and Applications, Shandong Normal University, No 88, East Wenhua Road, Jinan 250014, China; lnzhao@sdnu.edu.cn (L.Z.); 2019020530@stu.sdnu.edu.cn (Y.Y.); 2020010065@stu.sdnu.edu.cn (L.T.); 2020020563@stu.sdnu.edu.cn (W.Z.); 2020020556@stu.sdnu.edu.cn (Z.Z.); ningtingyin@sdnu.edu.cn (T.N.); 2 Shandong Provincial Key Laboratory of Optics and Photonic Device, No 88, East Wenhua Road, Jinan 250014, China; 3 School of Physical Science and Technology, Soochow University, Suzhou 215006, China

**Keywords:** pulsed laser, saturable absorber, nanoparticles

## Abstract

A stable, passively Q-switched Yb:CaGdAlO_4_ laser based on Mo:BiVO_4_ saturable absorber was demonstrated. Close observations of the structure and morphology of the nanoparticles by using transmission electron microscope, Raman spectrum and linear absorption were measured. The nonlinear transmission of Mo:BiVO_4_ was characterized by a 30 ps laser with a central wavelength of 1064 nm and a repetition rate of 10 Hz. The experimental maximum output power of the pulsed laser was 510 mW with a repetition rate of 87 kHz and pulse width of 3.18 μs, corresponding to a peak power of 1.84 W and a single pulse energy of 5.8 μJ. The experimental results indicate that Mo:BiVO_4_-SA is a great candidate for passively Q-switched lasers in the near infrared region.

## 1. Introduction

It has been well known that pulsed lasers have been applied in various areas, such as material processing, optical communication and medicine. The passively Q-switching technique via inserting saturable absorbers (SAs) in the resonators is a typical method to generate pulsed lasers [1,2,3,4,5]. Numerous studies on SAs have been done to investigate pulsed lasers. Relatively mature SA, GaAs-based semiconductor saturable absorber mirrors (SESAMs) have been extensively utilized for pulsed laser generation [6]. However, complicated fabrication procedures are required for SESAMs. Other SAs, including black phosphorus (BP) [7,8], carbon nanotubes (CNTs) [9,10] and two-dimensional (2D) layered materials [11,12,13,14], have been investigated for pulsed laser generation. Due to the flexible bandgap modulation, these materials show excellent performance over a broad spectrum ranging from the visible to mid-infrared, but there are still inherent drawbacks to them. BPs have the problem of long-term instability, CNTs are restricted by pipe diameter, and the low damage threshold of 2D materials limits their operability at higher power. Therefore, further effort needs to be made to search for new materials with better performance for pulsed laser generation.

Recently, bismuth vanadate (BiVO_4_) has widely been used in the fields of transportation services, medicine, and solar energy due to its proper band location, great stability, absence of toxic heavy metals and environment friendliness [15,16,17]. BiVO_4_ is also a type of semiconductor material. With accurate control of the parameters, it can be potentially applied as a saturable absorber. However, BiVO_4_ suffers from the challenging technical issue of the relatively low mobility of photogenerated charges, which hampers the separation of electron-hole pairs. The excited electron-hole pairs are well separated and the charge carrier mobility is greatly enhanced by doping with high valence metal Mo^6+^ or W^6+^, which is incorporated into the partial sites of V^5+^ in BiVO_4_. The conductivity and photocatalytic ability are greatly improved [18,19]. From the experimental data provided by Hoffart et al., the total conductivity of 5% Mo-doped BiVO_4_ was at least an order of magnitude higher than that of BiVO_4_ at 600 °C [20]. The band gaps of BiVO_4_ and Mo:BiVO_4_ are 2.43 and 2.42 eV, corresponding to absorption edge of 510 and 513 nm, respectively [21]. However, several optical characteristics, including nonlinear transmission, modulation depth, and saturable influence, have not been researched for laser modulators.

In this paper, Mo:BiVO_4_ nanoparticles were successfully prepared. Close observation of the structure and morphology of the nanoparticles by using transmission electron microscope (TEM), Raman spectrum and linear absorption were completed. The nonlinear saturable absorption of Mo:BiVO_4_ was characterized by a 30 ps pulsed laser with a central wavelength of 1064 nm and a repetition rate of 10 Hz. The modulation depth and saturation intensity were obtained. Combined with Mo:BiVO_4_-SA, an a-cut Yb:CaGdAlO_4_ (Yb:CALGO) was used as the laser gain medium to test pulsed laser operation at 1 μm. The Yb:CALGO is a competitive crystal for high-power laser diode pumping due to its excellent thermal conductivity (6.9 W m^−1^K^−1^) and extremely broad emission bandwidth ranging from 1030 to 1090 nm (0.75 × 10^−20^ cm^2^ for σ-polarization and 0.25 × 10^−20^ cm^2^ for π-polarization at 1040 nm) [22]. The experimental maximum output power of the pulsed laser was 510 mW, with a repetition rate of 87 kHz and a pulse width of 3.18 μs, corresponding to a peak power of 1.84 W and a single pulse energy of 5.8 μJ.

## 2. Materials and Methods

**Figure 1:**
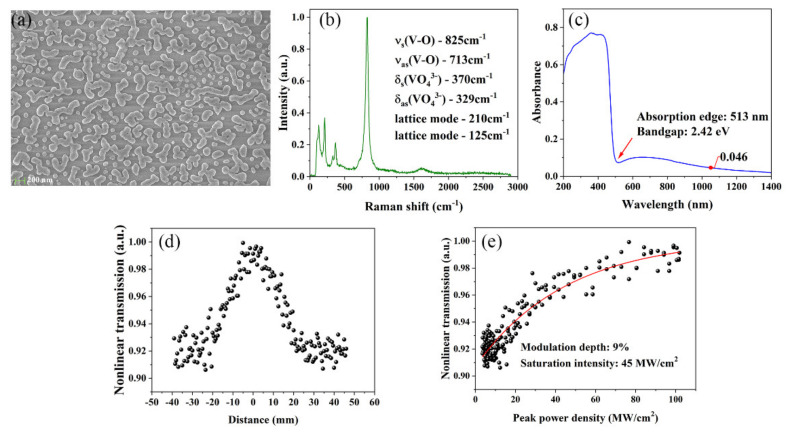
(**a**) The TEM image. (**b**) The Raman spectrum. (**c**) Absorptance of the Mo:BiVO_4_ film. (**d**) Z-scan curve of Mo:BiVO_4_-SA. (**e**) The dependence of nonlinear transmission on peak power density.

The preparation of BiVO_4_ thin film is improved by sol–gel and calcining [23,24]. First, bismuth nitrate pentahydrate was dissolved in glacial acetic acid and stirred for 30 min to form 0.2 M solution A. At the same time, a certain quantity of vanadium oxyacetylacetonate was dissolved in acetylacetonate solution and stirred for 1 h to form 0.2 M solution B. Then, acetylacetone molybdenum was dissolved in acetylacetone solution to form solution C with the same molar concentration as solution A. Mix the three solutions of A, B and C according to the metal molar ratio Bi:V:Mo = 1:0.99:0.01. The mixed solution was exposed to supersonic waves for 40 min, taken out and stood still for 6 h to obtain a 1% molybdenum doped bismuth vanadate precursor. Taking 380 μL of precursor liquid with a pipette gun, drop and apply it to the clean and dry quartz sheet (30.0 mm × 30.0 mm × 1.0 mm), spin it at a speed of 700 rpm for 10 s, put the quartz sheet with precursor liquid in the oven at 150 °C for 10 min and calcine it in the muffle furnace at 470 °C for 30 min. Carry out the above procedure twice. Mo:BiVO_4_ films were obtained. As shown in Figure 1a, the TEM image of the Mo:BiVO_4_ film indicates the nanoparticles are uniformly distributed.

The Raman spectrum of Mo:BiVO_4_ nanoparticles is shown in Figure 1b. The strongest band was 825 cm^−1^, the moderate band was 370, 329, 210 and 125 cm^−1^, and the weak band was 713 cm^−1^. The highest Raman band at 825 cm^−1^ belongs to the symmetric V-O stretching mode *υ*_s_(V-O), the band at 713 cm^−1^ belongs to the antisymmetric V-O stretching mode *υ*_as_(V-O), the band at 370 and 329 cm^−1^ are the symmetric *δ*s(VO_4_^3−^) and antisymmetric bending modes *δ*as(VO_4_^3−^), respectively, while the lattice modes occur at 210 and 125 cm^−1^. All of the Raman bands are in good accordance with the characteristic Raman peaks of Mo:BiVO_4_ [25].

Figure 1c shows the diffuse reflectance spectrum of Mo:BiVO_4_ film. The intense absorption is in the ultraviolet and visible region (from 200–513 nm), which is due to the transition at the band gap. The absorption edge is located at 513 nm, corresponding to the bandgap of 2.42 eV. However, there is still a non-negligible absorbance in the near-infrared range (>850 nm). For instance, the absorptance at 1050 nm is around 0.046, which is what eliminated the impact of the substrate. The main reason is the edge-state absorption in the region corresponding to sub-bandgap photon energies, which has been shown in other semiconducting materials, such as MoS_2_ [26,27,28]. The states at the band edges enable electrons to be excited to the edge-state levels within the material bandgap. The Pauli blocking effect could saturate this edge-induced sub-bandgap absorption of light at high intensities. In previous research, the indirect gap for few-layer MoS_2_ was 1.41 eV (879 nm), but few-layer MoS_2_ as a saturable absorber could still operate at 1566 and 1924 nm (sub-bandgap energies) to create pulsed lasers [26]. Therefore, Mo:BiVO_4_ film could potentially operate in the infrared region as a laser modulator.

The nonlinear transmission curve of Mo:BiVO_4_-SA was measured by the z-scan method using a mode-locked 1064 nm laser with a repetition frequency of 10 Hz and a pulse width of 30 ps, as shown in Figure 1d. The dependence of nonlinear transmission *T* on input peak power density *I* can be obtained according to the data of Figure 1d using the following Formula:

(1) $$T=1-\Delta D*\exp(-\frac{I}{I_{s}})-\alpha_{ns}$$

where Δ*D* is the modulation depth, *I_s_* is the saturation intensity, and α*_ns_* is non-saturable loss. As shown in Figure 1e, the modulation depth and saturation intensity of Mo:BiVO_4_-SA are 9% and 45 MW/cm^2^.

**Figure 2:**
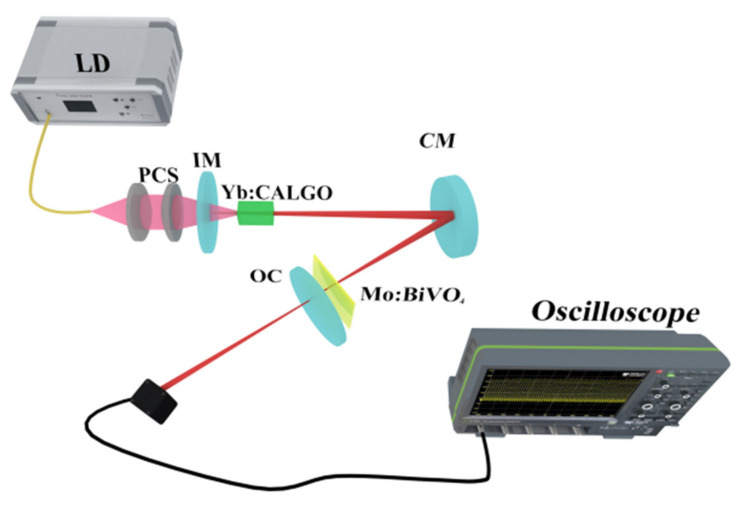
Draft of the CW and pulsed Yb:CALGO laser.

The draft of the continuous wave (CW) and pulsed Yb:CALGO laser is shown in Figure 2. The pump light is a 976 nm laser diode array with a maximum output power of 30 W and a core diameter of 100 μm. It is focused onto a Yb:CALGO crystal through a 1:1 collimating coupler. The dimension of a-cut Yb:CALGO (3 at.%) is 4 × 4 × 8 mm^3^. Both end surfaces of Yb:CALGO are antireflection-coated (R < 1%) at the 976 nm and 1030–1080 nm. A V-type laser cavity is utilized to research the pulsed laser. Both sides of the input mirror (IM) are antireflection-coated at 976 nm (R < 1%) and the right side is high reflectively (HR) coated (R > 99%) at 1030–1080 nm. The CM is a concave mirror whose curvature radius is 100 mm, and it is HR coated in the band 1030–1080 nm. As for the output coupler (OC), two plane mirrors with different transmissions of 1% and 2% at 1030–1080 nm are utilized, respectively. By calculation, the beam radii in the laser crystal and Mo:BiVO_4_-SA are 106 μm and 96 μm, according to the ABCD propagation matrix.

## 3. Results

**Figure 3:**
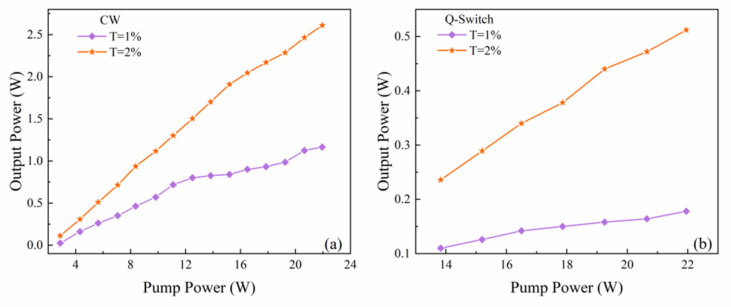
(**a**) The output power of CW Yb:CALGO laser. (**b**) The output power of pulsed Yb:CALGO laser using Mo:BiVO_4_-SA.

The resonator firstly operated at CW state without SA, and two types of OCs were used for comparison in a similar experimental setup. The output power reached the peak 1.17 W with a slope efficiency of 5.7% when the transmission of OC is 1%. To improve the output power, the OC was replaced with another one with T = 2%. The resonator reached a maximum output power of 2.6 W, with a slope efficiency of 13.2%. The pump power did not exceed 22 W to prevent the laser crystal from damage risk. The performances of CW operation are demonstrated in Figure 3a. The Mo:BiVO_4_-SA was then inserted into the resonator and it was placed close to OC. The laser could stably operate at passively Q-switched state by carefully adjusting the SA under the pump power of 13.8 W. The cavity delivered the maximum output power of 178 mW and 510 mW when the pump power reached 22 W, corresponding to the OC of T = 1% and 2%, respectively. From the comparison of Figure 3a,b, one can see that the output power of pulsed laser decreases a lot more than CW operation. The main reason is that the insertion of Mo:BiVO_4_-SA results in more loss to the resonator.

**Figure 4:**
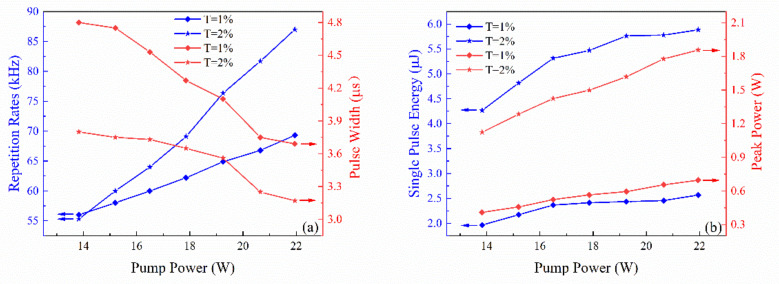
(**a**) The dependence of repetition rates and pulse widths on pump power. (**b**) The dependence of single pulse energy and peak power on pump power.

The dependence of repetition rate and pulse widths on pump power is shown in Figure 4. Both of them represent typical characteristics of a passively Q-switched laser: the repetition rate increases while the pulse width reduces when pump power increases. For T = 2% OC, when pump power reached 13.8 W, the pulse width was 3.83 μs with a repetition rate of 55 kHz. The pulse width reduced to minimum 3.18 μs while the pulse repetition rate increased to 87 kHz under the pump power of 22 W. For T = 1% OC, the pulse width reduced from 4.8 μs to 3.7 μs while the pulse repetition rate increased from 56 kHz to 69 kHz. The single pulse energy and peak power based on different transmissions of output couplers were calculated according to the experimental results, as shown in Figure 4b. The detailed data is shown in Table 1.

**Figure 5:**
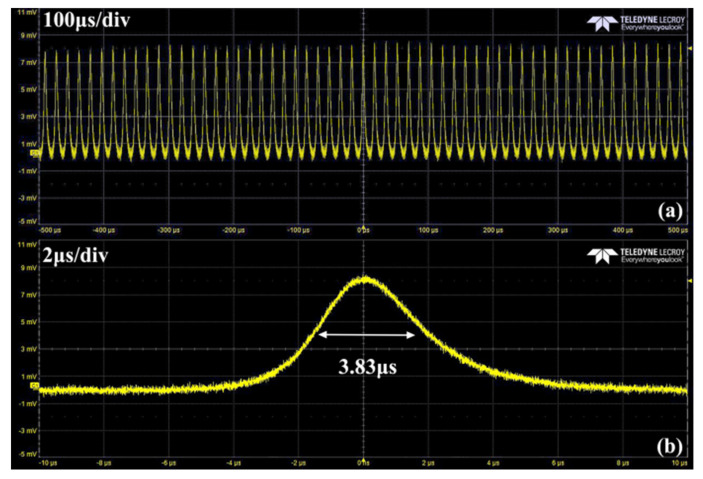
The pulse sequence (T = 2%) recorded at (**a**) 100 μs/div and (**b**) 2 μs/div.

From the data in Table 1, the operation of passively Q-switched laser with T = 2% OC is better than T = 1%. The pulse width is shorter and the output power is higher. Thus, in the following experiment, the OC with T = 2% was used to measure the pulse sequence, beam pattern and wavelength. A Si detector (Thorlabs, DET025AFC, Newton, NJ, USA) and a digital oscilloscope with a bandwidth of 1-GHz (LeCroy, HDO4104A, NY, NY, USA) were used to measure the pulsed laser performance and record the passively Q-switched pulse sequence. Figure 5 shows the pulse train at different time scales. The sequence at 1 ms time scale demonstrates the pulsed laser performed stably. It indicates that Mo:BiVO_4_ is a promising SA for pulsed laser generation.

**Figure 6:**
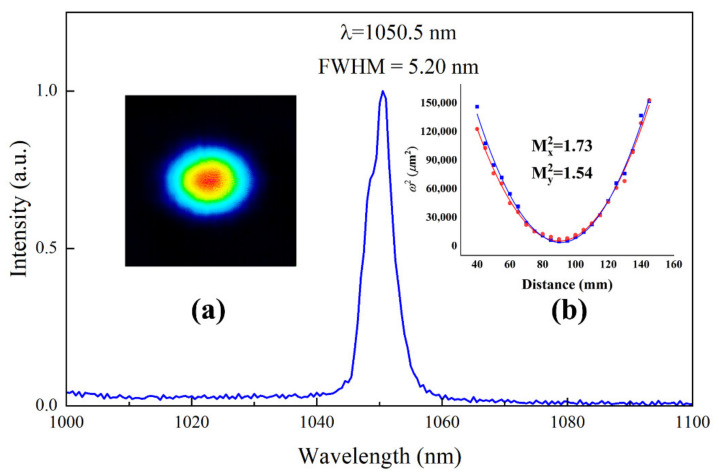
Pulsed laser spectrum. Insert (**a**): laser beam profile. Insert (**b**): M^2^ factor.

The beam profile was recorded by a CCD camera (Dataray, S-WCD-LCM-C-UV, CA, USA) when the laser was oscillating in Q-switched operation state, as shown in Figure 6a. The beam radii at different positions were measured and the M^2^ factors were fitted to be $M_{x}^{2}=1.73$ and $M_{y}^{2}=1.54$ (Figure 6b). The spectrum of the pulsed laser was measured by an optical spectrum analyzer (Avantes, AVASPEC-3648-USB2, Apeldoorn, Netherlands). The wavelength was 1050.5 nm and the full width at half maximum was 5.2 nm.

## 4. Discussion and Conclusions

In conclusion, Mo:BiVO_4_-SA was used to create a Q-switched laser with a high repetition rate for the first time. The nonlinear transmission curve of Mo:BiVO_4_ was measured experimentally. The modulation depth and saturation intensity of Mo:BiVO_4_-SA are 9% and 45 MW/cm^2^. The experimental results indicate that Mo:BiVO_4_-SA exhibits a high modulation depth and excellent stability. Although the visible bandgap of Mo:BiVO_4_-SA suggests the suitability of the laser modulator in the visible region, the edge-state induced absorption in the infrared indicates that Mo:BiVO_4_-SA is a great candidate for developing pulsed lasers in broadband. With Mo:BiVO_4_-SA as SA, the Q-switched laser delivered a maximum output power of 510 mW with a repetition rate of 87 kHz and pulse width of 3.18 μs, corresponding to a peak power of 1.84 W and a single pulse energy of 5.8 μJ. By changing the doping concentration, Mo:BiVO_4_-SA is expected to generate a shorter pulse by Q-switching or CW mode locking. Moreover, considering its excellent performance in the visible region, using Mo:BiVO_4_-SA to generate a visible pulsed laser is our next planned study.

## Figures and Tables

**Table 1:** Parameters of the pulsed Yb:CALGO laser under different OCs.

OC	Output Power (mW)	Repetition Rate (kHz)	Pulse Width (μs)	Single Pulse Energy (μJ)	Peak Power (W)
1%	178	69	3.7	2.6	0.7
2%	510	87	3.18	5.8	1.84
